# Detection of discriminative sequence patterns in the neighborhood of proline cis peptide bonds and their functional annotation

**DOI:** 10.1186/1471-2105-10-113

**Published:** 2009-04-20

**Authors:** Konstantinos P Exarchos, Themis P Exarchos, Costas Papaloukas, Anastassios N Troganis, Dimitrios I Fotiadis

**Affiliations:** 1 Unit of Medical Technology and Intelligent Information Systems, Department of Computer Science, University of Ioannina, Ioannina, Greece; 2 Department of Biomedical Technology, CERETETH, Larissa, Greece; 3 Department of Medical Physics, Medical School, University of Ioannina, Ioannina, Greece; 4 Department of Biological Applications and Technology, University of Ioannina, Ioannina, Greece

## Abstract

**Background:**

Polypeptides are composed of amino acids covalently bonded via a peptide bond. The majority of peptide bonds in proteins is found to occur in the *trans *conformation. In spite of their infrequent occurrence, *cis *peptide bonds play a key role in the protein structure and function, as well as in many significant biological processes.

**Results:**

We perform a systematic analysis of regions in protein sequences that contain a proline *cis *peptide bond in order to discover non-random associations between the primary sequence and the nature of proline *cis/trans *isomerization. For this purpose an efficient pattern discovery algorithm is employed which discovers regular expression-type patterns that are overrepresented (i.e. appear frequently repeated) in a set of sequences. Four types of pattern discovery are performed: i) exact pattern discovery, ii) pattern discovery using a chemical equivalency set, iii) pattern discovery using a structural equivalency set and iv) pattern discovery using certain amino acids' physicochemical properties. The extracted patterns are carefully validated using a specially implemented scoring function and a significance measure (i.e. log-probability estimate) indicative of their specificity. The score threshold for the first three types of pattern discovery is 0.90 while for the last type of pattern discovery 0.80. Regarding the significance measure, all patterns yielded values in the range [-9, -31] which ensure that the derived patterns are highly unlikely to have emerged by chance. Among the highest scoring patterns, most of them are consistent with previous investigations concerning the neighborhood of *cis *proline peptide bonds, and many new ones are identified. Finally, the extracted patterns are systematically compared against the PROSITE database, in order to gain insight into the functional implications of *cis *prolyl bonds.

**Conclusion:**

*Cis *patterns with matches in the PROSITE database fell mostly into two main functional clusters: family signatures and protein signatures. However considerable propensity was also observed for targeting signals, active and phosphorylation sites as well as domain signatures.

## Background

**Figure 1:**
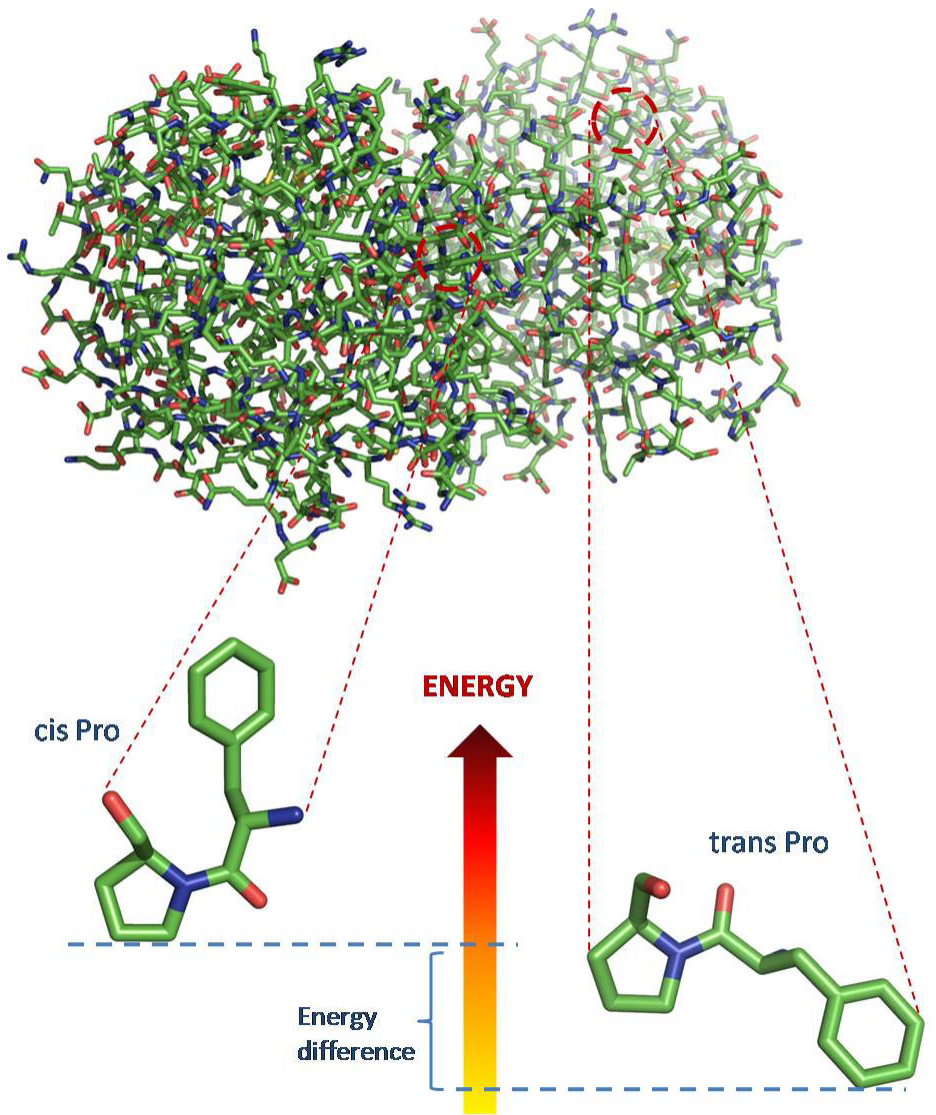
**Conformational isomers of a Phenylalanine-Proline (Phe-Pro) peptide bond. The displayed structure is 2H50, obtained from the Protein Data Bank**. The zoomed isomers are indicative of the differences in the three dimensional structure of a Phe-Pro peptide bond in *cis *and in *trans *conformation respectively. In the *cis *conformation, the alpha carbons are locked on the same side of the peptide bond, whereas in the *trans *conformation they lie on opposite sides relative to the bond. Moreover in the lower part of the figure, a schematic representation of the energy difference between the two conformations is shown.

The peptide bond linking adjacent amino acid residues in a protein backbone can adopt either the *cis *or *trans *conformation. The *cis *conformation occurs rarely in polypeptides because of the higher intrinsic energy compared to the *trans *conformation (Figure 1) [1]. However, in the case of X-P (where X denotes any of the 20 amino acids and P is Proline) amino acid pairs, the situation is slightly different, since the free energy difference between the *cis *and *trans *isomers is much smaller. In fact, a survey conducted by Weiss *et al*. [2], reported that 0.03% of the X-nonP (where nonP denotes any amino acid except Proline) and 5.2% of the X-P peptide bonds are in *cis *conformation.

*Cis *peptide bonds are very important in a variety of biological processes. The isomerization of proline bonds is catalyzed by the peptidyl prolyl isomerases (PPIases), which are also implicated in a variety of severe diseases. Recent studies [3] have indicated that prolyl *cis/trans *isomerization can act as a molecular timer, to help control the amplitude and duration of a cellular process, making it a new target for therapeutic interventions. Moreover, *cis *prolyl residues are more often conserved than the surrounding amino acids, which show the same extent of conservation as the whole protein, indicating the significance of *cis *prolyl bonds in protein structure and function during evolution [4]. Furthermore *cis *peptide bonds are located near the active sites of proteins, or have roles in the function of the protein molecules [5]. In addition proline *cis/trans *isomerization is known to play a critical role in protein folding, splicing, cell signaling and transmembrane active transport [6].

Proline isomerization is also inherently related to regions of proteins replete with proline. These proline rich regions (PRRs) occur frequently both in prokaryotes and eukaryotes and play important roles in protein-protein interactions or participate in important structural elements. In particular, SH3, WW and several new protein-interaction domains prefer ligand sequences that are rich in proline. The relative rigidity of PRRs allows for weaker interactions, thus facilitating their binding variability and versatility in signaling pathways [7]. Another distinctive characteristic of the proline residue refers to its puckering preference with respect to the conformation of the preceding peptide bond [8,9]. Specifically, prolines in *cis *conformation preferentially adopt a down puckering, thus achieving attenuation of the severe steric hindrance between the proline and the preceding residue. On the other hand, prolines in *trans *conformation are almost evenly found with either up or down puckering [10].

Several factors have been reported in the literature to affect the conformation of the peptide bond. Nuclear Magnetic Resonance (NMR) experiments have shown that the *cis/trans *isomerization of proline peptide bonds is not a strictly localized event, but is significantly influenced by the amino acid sequence adjacent (preceding and succeeding) to the proline (i.e. the determination of the peptide bond conformation is encoded in the amino acid sequence) [11]. This observation has been further reinforced by automatic methods aiming to predict the peptide bond formation mostly in X-P amino acid pairs [12-14] or between any two amino acids in general [15-17]. These studies base their predictions either solely on the amino acid sequence, or extract several characteristics (secondary structure, physicochemical properties, etc.) from the sequence in order to predict the conformation of the peptide bond. It should be noted that these characteristics span to a certain extent in the sequence and do not concern individual amino acids. Hence, it is clear that the conformation of the peptide bond between two residues is encoded in the amino acid sequence, but also that certain non-random patterns in the sequence are involved and influence the formation of the peptide bond. Thus, an analysis which is able to identify and evaluate these patterns will provide insight towards the mechanism of the peptide bond formation.

*Cis/trans *isomerization has attracted increased interest over the last years [3,4,13-19]. The majority of the methods proposed in the literature aiming to predict the *cis/trans *isomerization are based on machine learning schemes, whereby a classifier is trained to distinguish between the two conformations, without providing sufficient insight related to the nature and the mechanism of the peptide bond. Frömmel *et al*. [12] extracted six patterns, based on the physicochemical properties of the amino acids, in order to discriminate the two conformations of prolyl peptide bonds. However, this method was developed using a rather small dataset (242 X-P bonds), thus diminishing the credibility and generality of the proposed patterns, which were later tested on a larger dataset, yielding unsatisfactory results. Wang *et al*. [13] employed only the primary amino acid sequence, coded in binary form in order to predict the proline isomerization. Pahlke *et al*. [15] developed an algorithm, based on the Chou-Fasman parameters, in order to predict the peptide bond conformation using as input the secondary structure of amino acid triplets. Song *et al*. [14] employed multiple sequence alignment profiles coupled with secondary structure information aiming to predict the conformation of proline peptide bonds. Exarchos *et al *[17]. utilized a large feature vector comprising of multiple sequence alignment profiles, secondary structure information, solvent accessibility and the physicochemical properties of the neighboring amino acids in order to predict the peptide bond conformation between any two amino acids.

In this work we perform a systematic analysis of regions containing *cis *prolyl peptide bonds for occurrences of non-random patterns which are associated with segments of X-P *cis *peptide bonds and accurately describe the nature of these bonds. We efficiently detect all maximal patterns (i.e. patterns that can not be made more specific without simultaneously affecting their length or composition) of variable length in a set of protein sequences. Among the amino acids, conservative substitutions are assumed concerning the structural or chemical nature of the residues, or individual physicochemical properties such as charge, hydrophobicity, etc. These regular expression-type patterns provide simple and understandable descriptors of *cis *prolyl containing segments, revealing structural and physicochemical characteristics that govern the peptide bond formation. Patterns of this type have been previously employed to describe other significant protein regions [20-23]. Besides the biological insight, the derived patterns could also be helpful in improving currently available prediction methods; this could be achieved by applying the extracted patterns as a pre- or post-processing stage in order to either refine the regions to search for *cis *peptide bonds or filter out certain overpredictions [24]. Furthermore, the functional propensity of the derived *cis *patterns is analyzed by exploiting the information deposited in the PROSITE database [25]. The methodological framework proposed in the present study constitutes a rather generic procedure with twofold contribution: first, we present an orchestrated approach for the extraction of overrepresented patterns in a set of sequences, allowing certain clusterings with biological insight among the amino acids. Subsequently, the extracted patterns are carefully distilled yielding a list of highly selective patterns, which accurately describe significant protein regions. Next, we present an efficient and systematic approach for the functional annotation and extrapolation of uncharacterized protein motifs.

## Methods

**Figure 2:**
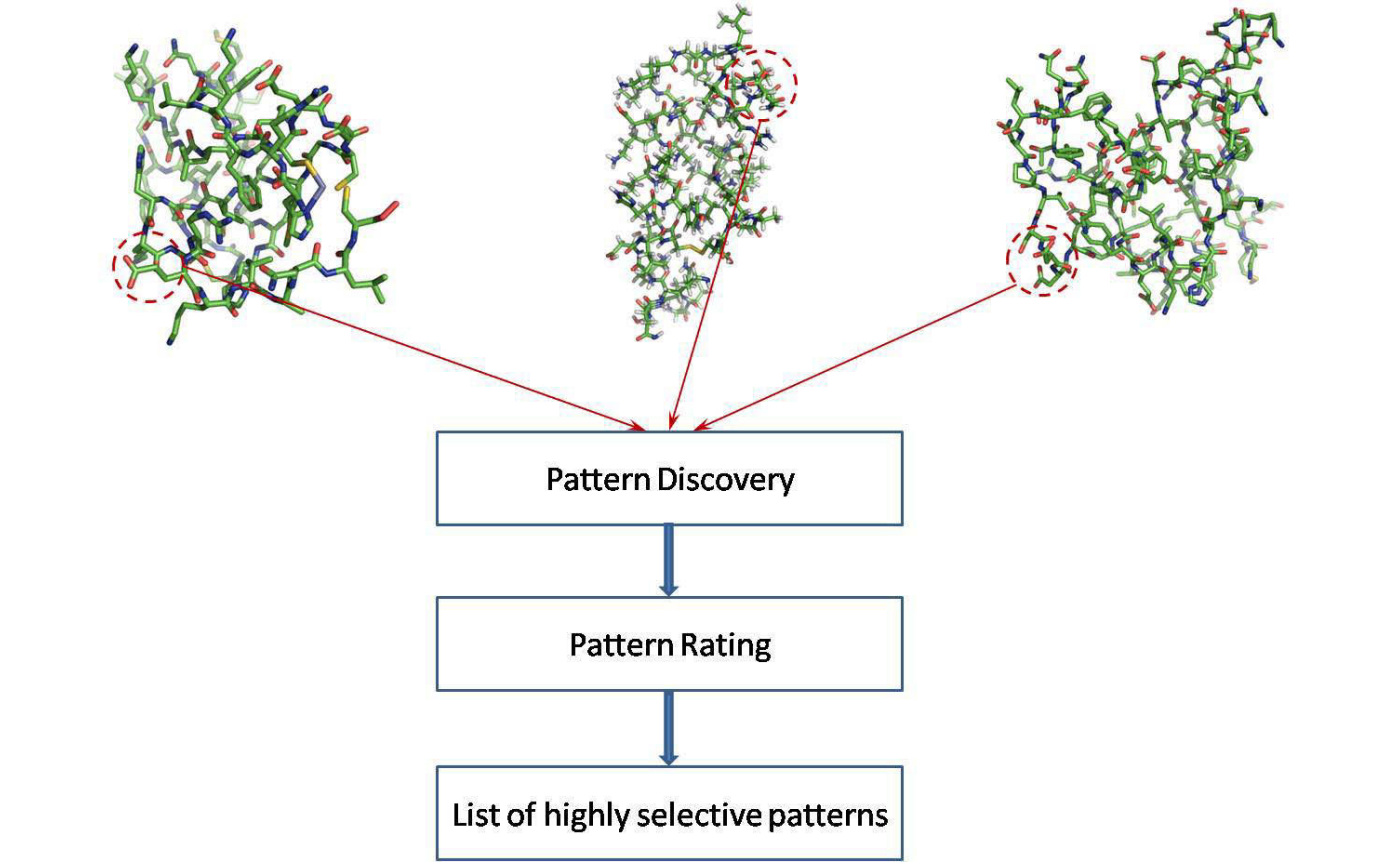
**Overview of the proposed analysis**. From the initial set of protein sequences, all regions containing a proline peptide bonds in *cis *conformation are assembled and representative patterns for these regions are extracted. Then, the pattern rating procedure discards superficial patterns of low selectivity, maintaining a list of highly descriptive patterns.

The overview of the proposed analysis is presented in Figure 2. First, the regions containing a *cis *proline peptide bond are isolated. Next, an efficient pattern discovery algorithm searches across these regions for regular expression-type patterns that are overrepresented in the neighborhood of proline *cis *peptide bonds. Then, during the pattern rating stage, the extracted patterns are carefully rated and only a list of highly selective patterns is retained.

### Dataset

The employed dataset includes 3050 high quality protein sequences obtained from the Protein Data Bank (PDB) [26] using the Protein Sequence Culling server (PISCES) [27] as a front-end interface for accessing and mining the PDB repository. These structures have been determined by X-ray crystallography to a resolution of 2.0Å or better and R-factor less than 0.25. The obtained sequences are related to one another by no more than 25% sequence identity. The annotation of the dataset was performed using the Volume Area Dihedral Angle Reporter (VADAR) [28], which accepts PDB [26] formatted files and calculates the dihedral angle ω among many other structural parameters. A peptide bond is considered to be in *cis *conformation if the ω angle was between -30 and +30, whereas bonds with angles outside of this range are assumed to be in *trans*.

Among the total 3050 protein sequences in the current dataset, there exist 32085 X-P peptide bonds, from which 1417 are in *cis *and 30668 in *trans *conformation. For each of these bonds a region of 11 amino acids length was formed by taking into account the ± 5 neighboring residues [14,17]; outside this range, the influence of the surrounding residues towards the peptide bond conformation decreases. Thus, two datasets were assembled: *D*_*cis *_containing 1417 *cis *proline regions and *D*_*trans *_containing 30668 *trans *proline regions. All regions have the same length (i.e. 11 residues) and are aligned to the proline residue in the center. Prolines situated at the beginning or the end of the protein sequences, which do not have adequate number of neighbors, were excluded from our study.

### Pattern discovery

**Figure 3:**
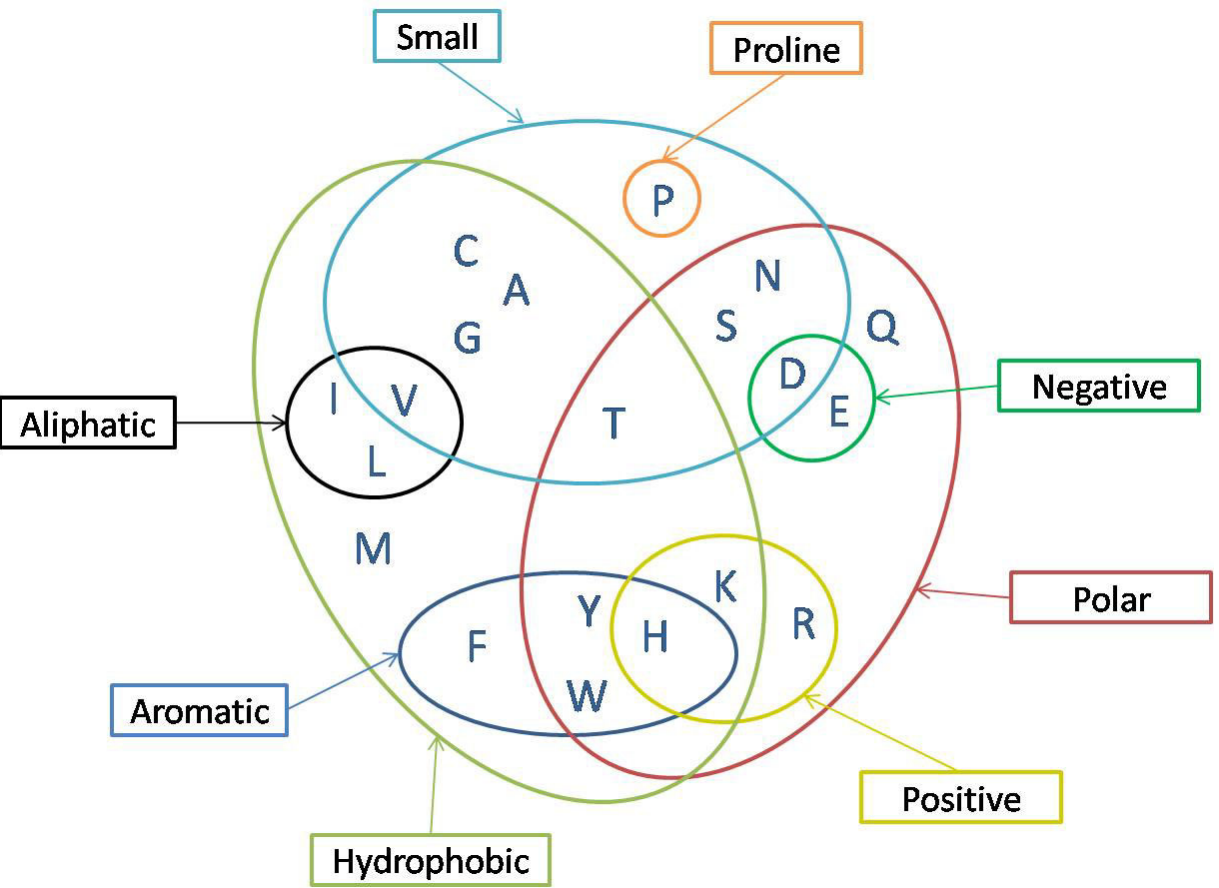
**Venn diagram representing the relationship of the 20 naturally occurring amino acids to a set of common physicochemical properties**. Besides common attributes of the amino acids such as hydrophobicity, size or charge, proline was also included in our study because of its unique backbone properties.

Our main aim is to extract regular expression-type patterns which are representative of the *cis *proline regions and help us identify factors with biological insight, encoding or affecting the formation of *cis *proline bonds. For this purpose the *D*_*cis *_was properly prepared and provided as input to the TEIRESIAS pattern discovery algorithm [29]. TEIRESIAS is able to rapidly identify all maximal patterns in a set of sequences, but additionally features some capabilities specifically tailored for biological sequences. TEIRESIAS operates in two phases: scanning and convolution, avoiding the enumeration of the entire solution space. During the scanning phase, patterns exceeding a minimum support threshold are maintained; then these elementary patterns are progressively combined into larger patterns, until all the existing maximal patterns are discovered. The reported patterns are guaranteed to be maximal such that any reported pattern can not be made more specific and still keep on appearing at the exact same positions within the input sequences. Moreover, TEIRESIAS requires a set of user-specified parameters which express the features of the extracted patterns. Specifically L corresponds to the minimum number of literals (i.e. non-wild-characters) in any pattern of the discovered patterns; W denotes the maximum extent of an elementary pattern, i.e. the maximum extent spanned by L consecutive (not contiguous) literals in the reported patterns; and K is the minimum acceptable support for a pattern in the specified input. In this study L was set equal to 3 as it has been shown to be the minimum value for which the convolution stage of the TEIRESIAS algorithm successfully operates during the pattern discovery process [29]. The value of W was chosen to be equal to 11, which is the length of all regions provided as input. Values larger than this would not be rational whereas smaller values would unreasonably restrict the span of the reported patterns. The minimum allowed support K for all reported patterns was set equal to 2 [29,30]. Since the extracted patterns are carefully validated during the pattern rating stage, an initially small threshold was chosen for the support. Four types of pattern discovery were carried out according to the permitted amino acid equivalencies, namely: i) exact pattern discovery, ii) pattern discovery assuming conservative replacement of chemically equivalent amino acids by one another: [AG], [DE], [FY], [KR], [ILMV], [QN], [ST] iii) pattern discovery using a structural equivalency set: [CS], [DLN], [EQ], [FHWY], [ITV], [KMR] and iv) pattern discovery allowing substitutions among amino acids belonging to a certain physicochemical property. The physicochemical properties employed in the last type of pattern discovery as well as the distribution of the amino acids among these properties are shown in Figure 3.

Based on Figure 3, for each physicochemical property two categories can be formed by checking whether a specific residue shares a certain property or not. For example, as far as polarity is concerned, the amino acids can be divided into two groups, one containing all amino acids with a polar or polarizable group, and another one containing the remaining amino acids. In a similar manner, equivalency sets can be formed for all properties in Figure 3.

The incorporation of such equivalencies along with the careful assessment of the derived patterns after each type of pattern discovery might provide some insight into the physical reasons for the occurrence of *cis *prolyl peptide bonds. Sequence patterns containing single amino acids or groups of amino acids, derived from the first three types of pattern discovery, are called *amino acid patterns*, whereas those composed of physicochemical properties (i.e. from the fourth type of pattern discovery), are called *property patterns*.

### Pattern rating

In order to further guarantee the specificity and selectivity of the derived patterns, we compare them with negative control sets. Especially, in our case where the classes are highly unbalanced (1417 *cis *and 30668 *trans*), the extracted patterns must be further rated in order to maintain patterns which accurately describe *cis *regions and match relatively few *trans *regions. The pattern rating is based on comparing, proportionally, the number of regions recovered by a pattern in the *D*_*cis *_and the *D*_*trans *_datasets. If *P *is a pattern and *M*(*P*) is the set of regions matching *P*, the scoring function can be defined as [22]:

(1)

where |*D*_*cis *_∩ *M*(*P*)| is the number of *cis *regions recovered by the pattern *P *in *D*_*cis*_, |*D*_*trans *_∩ *M*(*P*)| is the number of *trans *regions recovered by the pattern *P *in *D*_*trans *_and *balance *is the ratio of *cis *regions to *trans *regions; specifically in our case balance = |*D*_*cis*_|/|*D*_*trans*_| ≅ 0.05.

We introduce the variable *balance *in equation (1) because the two datasets are highly unbalanced and it is reasonable to expect |*D*_*cis *_∩ *M*(*P*)| and |*D*_*trans *_∩ *M*(*P*)| to be roughly proportional to the database size. In this way, we efficiently cope with the class imbalance problem without screening any potentially valuable negative examples or replicating examples from *D*_*cis*_. It is obvious that *Score *ranges in [0, 1], with 0 meaning that the respective pattern recovered only *trans *regions and 1 showing that a pattern is observed only in *cis *regions without matching any *trans *regions. A score threshold equal to 0.5 would exclude patterns that are either observed more frequently in *trans *regions or random patterns with no preference for *cis *or *trans *conformations. Since our aim is to deduce highly descriptive patterns that accurately describe the nature of *cis *regions a much more rigorous threshold for the score is needed. Specifically for the amino acid patterns the score threshold is set equal to 0.90, whereas, for the property patterns the threshold is slightly relaxed, and set equal to 0.80, to ensure sufficient number of retained associations. Property patterns occur more frequently in the database, and are more uniformly distributed between the two conformations; thus, lower scores are expected. However, both score thresholds were chosen after many experiments in order to ensure that a sufficient number of reliable associations is retained. Moreover, a further constraint is imposed on the derived patterns, which applies to both the amino acid patterns and property patterns:

(2)

The above condition requires that in case no matches are found in *D*_*trans*_, the number of matches in *D*_*cis *_should exceed a certain threshold as well. This guarantees that a pattern matching *D*_*trans *_zero times and *D*_*cis *_just once, yielding *Score *= 1, should by no means be identified as being highly descriptive. This threshold is slightly stricter in the case a pattern matches regions both in *D*_*cis *_and *D*_*trans*_. The choice of the thresholds is based on thorough inspection of the derived patterns as well as their relative matches in *D*_*cis *_and *D*_*trans*_. The combination of the scoring function and the above condition ensures that the maintained patterns are non-random and show strong preference for *cis *regions but also poor correlation with *trans *regions. Furthermore, a measure of significance is computed and attached to each pattern. This measure is estimated using the Bayes theorem in conjunction with a second order Markov chain by assuming that each of the patterns will be used as a predicate to search a database which has the size and composition of GenPept [31]. The reported values are the logarithms of the estimated probability of the pattern under consideration, and represent the possibility that the pattern under consideration is found by chance in a very large biological database.

### Functional analysis

**Figure 4:**
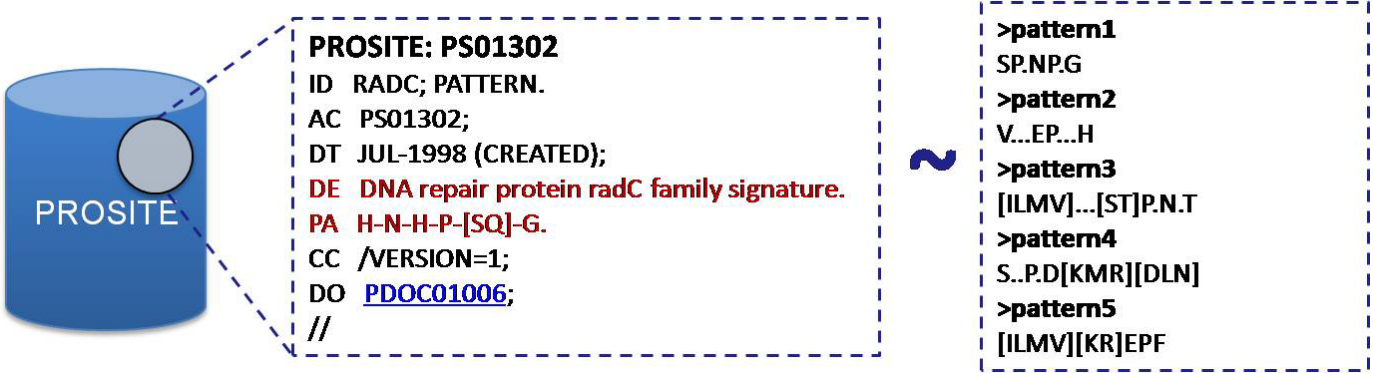
**Comparison of the derived patterns against the PROSITE database**. On the left we can see a typical PROSITE entry, where the Description line (DE) and the Pattern line (PA) are highlighted in red. The Description line contains functional annotation for the respective pattern on the Pattern line. On the right we can see some indicative *cis *patterns.

In order to gain some perspective about the functional implication of *cis *proline peptide bonds, we systematically search for overlaps between the *cis *patterns and the PROSITE patterns, and vice-versa (Figure 4). PROSITE is a major repository containing protein patterns grouped according to particular shared functional attribute, and has been carefully scrutinized, in order to omit redundant patterns. However, PROSITE does not contain a distinct category for *cis *regions, which have been thoroughly explored and mapped against its records, using the proposed analysis.

Initially, for the purposes of our study, a Perl script was implemented in order to achieve unanimity between the different conventions used for the regular expressions of the patterns. Then, every pattern from the PROSITE database is compared against all *cis *patterns. If an overlap is detected then we infer that there is correlation between the *cis *pattern and the function assigned in the Description line of the respective PROSITE record. This correlation is further supported by the lack of redundancy both in the PROSITE database and in the list of *cis *patterns. During the pattern comparison procedure, all residues are treated in the same manner and no restrictions are imposed, as long as an exact match is detected. Between two patterns all possible alignments are considered, essentially leading to an exhaustive search.

## Results and discussion

Using the above described methodological analysis, several regular expression-type patterns that accurately describe regions containing *cis *proline bonds are identified. The complete list of these patterns is available through a web server . Here only a summary of the most descriptive ones is presented. As mentioned above, two types of patterns are extracted, amino acids patterns and property patterns.

### Amino acid patterns

Table 1 summarizes the Top-20 amino acid patterns, which are obtained from the first three types of pattern discovery, after sorting the discovered patterns by score.

**Table 1:** The 20 highest scoring amino acid patterns.

**Exact pattern discovery**			**Chemical equivalency set**			**Structural equivalency set**		
**Pattern**	**Score**	**Significance**	**Pattern**	**Score**	**Significance**	**Pattern**	**Score**	**Significance**

FE.P...F	1	-15.53	[ST]P.NPTG	1	-24.47	[DLN]. [DLN]... [KMR]. [ITV] [CS]	1	-15.21

SP.NP.G	0.99	-19.82	[ST]PNNP.G	1	-25.08	[CS]..NPTG	1	-19.71

V...EP...H	0.99	-15.40	[ILMV]..P.NP.G	1	-18.46	SP.NP.G	0.99	-19.82

GPY.G	0.99	-14.93	[ST]PN..T [AG]	1	-18.51	[CS]... [FHWY]. [FHWY].N	0.99	-12.60

G...GPY	0.98	-15.08	[ST]P.NP.G	0.99	-18.81	[EQ].P [FHWY] [ITV].V	0.99	-17.76

P.NPTG	0.98	-19.68	[ILMV] [KR]EP [FY]	0.99	-17.54	[FHWY]P.E [FHWY]I	0.99	-19.11

PNNP.G	0.98	-20.06	SP.NP.G	0.99	-19.82	[ITV]..P.NP.G	0.99	-18.80

PY..SG	0.98	-14.71	[ILMV] [KR]EPF	0.99	-17.91	GPY.G	0.99	-14.93

NNPT	0.97	-14.60	[ILMV].... [QN]P.G	0.99	-12.62	[CS].PNNP	0.99	-19.63

V.....N..T	0.97	-8.11	[QN]..FV [FY]	0.99	-13.97	V...EP...H	0.99	-15.40

P..YP.K	0.97	-15.59	E.GP [FY]	0.99	-14.82	[ITV].SP. [DLN]P	0.99	-17.67

N...P.PE	0.97	-14.86	GPY.G	0.99	-14.93	PY.. [CS]G	0.98	-14.52

GPY...G	0.97	-15.25	PNNP [ST]	0.99	-19.40	P.NPTG	0.98	-19.68

SP.N..G	0.97	-14.40	[ILMV]. [ST].N...G	0.99	-12.25	G...GPY	0.98	-15.08

S..NP.G	0.96	-14.26	GY.. [ILMV].K	0.99	-13.20	PNNP.G	0.98	-20.06

T...NP.G	0.96	-14.38	C [ST]..NP	0.99	-14.33	[ITV] [FHWY]E...F	0.98	-13.02

PYG.S	0.96	-15.07	V...EP...H	0.99	-15.40	PY..SG	0.98	-14.71

QL.......Y	0.96	-9.12	[QN] [ST]P.N. [ST]	0.99	-17.92	S..P.D [KMR] [DLN]	0.98	-18.12

R...Y.P	0.96	-9.35	[ILMV]... [ST]P.N.T	0.99	-17.30	K.P [FHWY]T.. [ITV]	0.98	-18.59

N.K..F	0.96	-9.03	P [FY]PE. [AG]	0.99	-19.53	[KMR]..GP [FHWY].. [ITV]	0.98	-18.21

In the patterns presented in Table 1, some common conventions of regular expressions are observed. The dot "." is used to denote a position that can be occupied by an arbitrary residue, meaning that no considerable propensity for a certain amino acid is observed at the specified position. Also, square brackets " []", called "character classes" or "character sets", represent equivalency among the residues they contain.

In order to gain some qualitative conclusions about the nature of *cis *prolyl peptide bonds, the Top 20 patterns shown in Table 1, which contain several highly distinctive features of *cis *proline regions, are analyzed. In general, we can see that patterns derived using equivalency sets achieve slightly higher scores than exact pattern discovery. This indicates that certain structural and chemical properties of the amino acids contribute towards the discrimination between the two conformations. Although during exact pattern discovery the obtained patterns are stricter, thus fewer matches are expected for both types of peptide bonds, this does not justify lower scores, since the score for every pattern represents the ratio of matches in the *cis *and the *trans *regions dataset. Therefore, the lower score observed after performing exact pattern discovery, compared to the other two types of pattern discovery, shows that the ratio of matches in the two datasets, *D*_*cis *_and *D*_*trans*_, slightly changes in favor of the *trans *regions.

Furthermore, many patterns are common either completely unaltered or with small modifications in all three types of pattern discovery. These patterns are variations of the same underlying, yet highly descriptive pattern. For example "SP.NP.G", "GPY.G" and "V...EP...H" are obtained in each type of pattern discovery, and all of them yield very high scores, suggesting highly specific descriptors of *cis *regions. In addition slight variations of these patterns exist in all types of pattern discovery; in Table 2 the variations of the pattern "SP.NP.G", which exist only in the Top 20 patterns are shown. The patterns are aligned to one another and are grouped according to the type of pattern discovery. It should be noted that all these are maximal and the obtained scores at the pattern validation procedure are high, as it is shown in Table 1.

**Table 2:** Variations of the prevailing pattern "SP.NP.G".

**Exact pattern discovery**					S	P	.	N	P	.	G
	
						P	.	N	P	T	G
	
						P	N	N	P	.	G
	
							N	N	P	T	
	
					S	P	.	N	.	.	G
	
					S	.	.	N	P	.	G
	
				T	.	.	.	N	P	.	G
**Chemical equivalency set**					[ST]	P	.	N	P	T	G
	
					[ST]	P	N	N	P	.	G
	
			[ILMV]	.	.	P	.	N	P	.	G
	
					[ST]	P	N	.	.	T	[AG]
	
					[ST]	P	.	N	P	.	G
	
						P	N	N	P	[ST]	
	
			[ILMV]	.	[ST]	.	N	.	.	.	G
	
				[QN]	[ST]	P	.	N	.	[ST]	
	
	[ILMV]	.	.	.	[ST]	P	.	N	.	T	

**Structural equivalency set**					[CS]	.	.	N	P	T	G
	
			[ITV]	.	.	P	.	N	P	.	G
	
				[CS]	.	P	N	N	P		
	
			[ITV]	.	S	P	.	[DLN]	P		
	
						P	.	N	P	T	G
	
						P	N	N	P	.	G

**Figure 5:**
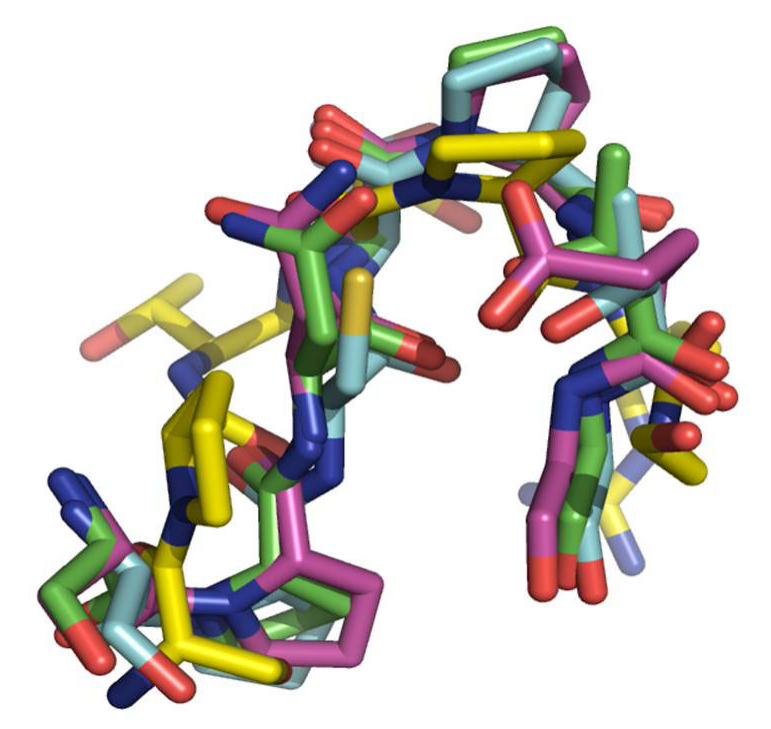
**Structural alignment of the regions containing the predominant pattern "SP.NP.G"**. The regions of the proteins matching with the "SP.NP.G" pattern were isolated, and then aligned by minimizing the RMSD (Root Mean Square Deviation) between the aligned residues.

Moreover, a structural alignment of the regions containing the high scoring pattern "SP.NP.G" is presented in Figure 5, to gain some perspective about the spatial characteristics and the morphology of this pattern.

**Figure 6:**
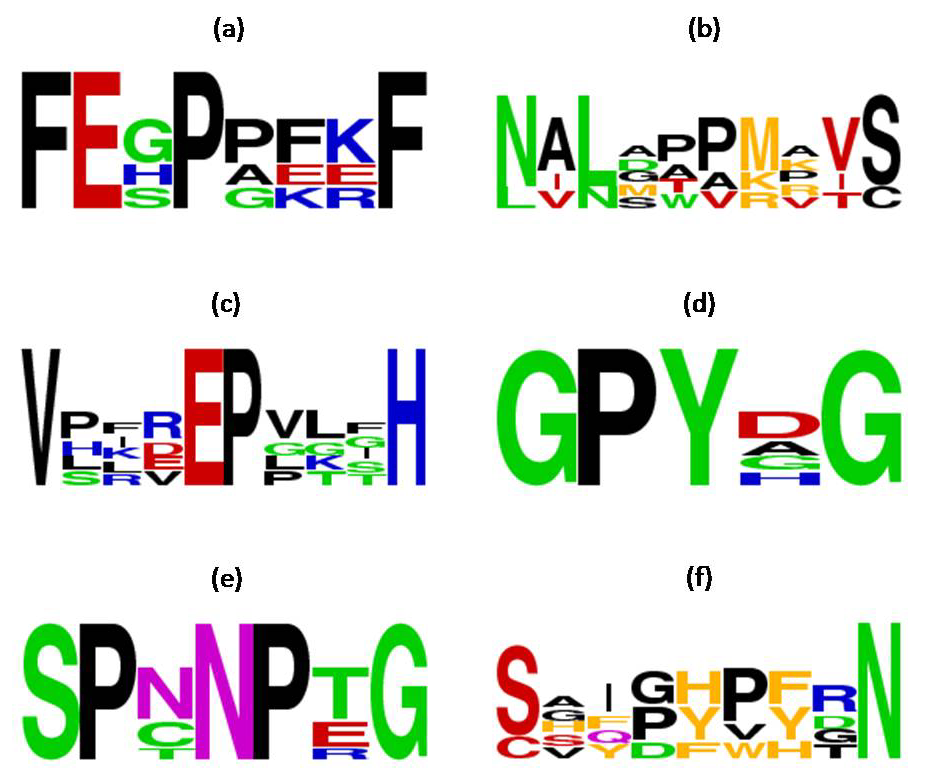
**Sequence logos for some representative amino acid patterns**. Specifically the following patterns are shown: (a) "FE.P...F", (b) " [DLN]. [DLN]... [KMR]. [ITV] [CS]", (c) "V...EP...H", (d) "GPY.G", (e) "SP.NP.G" and (f) " [CS]... [FHWY]. [FHWY].N".

Assessing the 20 highest scoring patterns, several interesting remarks can be deduced. Short polar residues, especially Serine (S) and Aspargine (N) are usually found as constituents of the *cis *peptide bond. This is mostly observed in the pattern "SP.NP.G" and its variations where the *cis *bond occurs frequently both between S-P and N-P. This observation is also consistent with the findings reported in [5] and [12]. Moreover Glycine (G) is found with high frequency either as part of the peptide bond or in its neighborhood. In Table 1, Glycine exists in 33 out of the 60 highest scoring patterns forming the neighborhood of *cis *proline bonds. Some representative patterns are "SP.NP.G" and its variations, "GPY.G", "G...GPY", " [KMR]..GP [FWHY].. [ITV]" and "PY..SG", to name a few. The tiny Glycine residue raises the probability of acquiring a *cis *conformation, probably due to the lack of steric hindrance. Several authors have highlighted the high frequency of Glycine in the immediate neighborhood of *cis *peptide bonds [1,4,5,11,12,18]. It has also been proven that Glycines, in contrast with positively charged residues, stabilize the *cis *conformation when found in positions succeeding the peptide bond. It has also been proposed in the literature that aromatic residues (Phenylalanine, Tryptophan, Histidine and Tyrosine) are frequent near *cis *conformation. This propensity is also confirmed in the discovered patterns; however, it is obvious that Tyrosine (Y), and Phenylalanine (F) to a smaller extent, are more frequent than the other two aromatic residues. "FE.P...F", "GPY.G", "PY..SG", " [ILMV] [KR]EP [FY]" and " [CS]... [FWHY]. [FWHY].N" are some of the highest scoring patterns that contain aromatic residues. Similar observations were made in [4,5], although in [4] a higher propensity of Tyrosine and Phenylalanine in the residues succeeding the X-P peptide bond is observed, thus reinforcing our observation. Furthermore, we can see a considerably high frequency of b-branched amino acids (Valine, Isoleucine and Threonine) in the neighborhood of *cis *peptide bonds. Some representative patterns are: "V...EP...H", " [DLN]. [DLN]... [KMR]. [ITV] [CS]", " [EQ].P [FHWY] [ITV].V" and " [ITV]..P.NP.G". According to [5] this observation is suggestive of the steric requirement for the isomerization of a *trans *peptide bond into *cis*. It is noteworthy that regions around *cis *formations are depleted in Alanine (A) and Aspartic acid (D). Alanine, is scarcely observed in the neighborhood of *cis *peptide bonds, and only in conjunction with the much more frequent Glycine (as part of the [AG] character class). In the case of Aspartic acid, we can see that it is apparent in three of the highest scoring patterns (" [DLN]. [DLN]... [KMR]. [ITV] [CS]", " [ITV].SP. [DLN]P", "S..P.D [KMR] [DLN]"), whereas only when the structural equivalency set is employed. Neither, during the exact patterns discovery, nor when the chemical equivalency set is employed does Aspartic acid appear as a constituent of any pattern. Hence, its appearance in the above patterns is mostly attributed to the character class it belongs (i.e. [DLN]). Some indicative patterns from Table 1 are also depicted as sequence logos in Figure 6, for a more intuitive and graphical representation [32].

### Property patterns

During the fourth type of pattern discovery, different sets of patterns are obtained, depending on the physicochemical property under consideration. Thus, according to Figure 3, eight sets of property patterns are elicited. The enumeration of the discovered patterns, along with the careful inspection of their physical meaning yields several interesting remarks. In Table 3 we can see the Top-10 property patterns discovered for each physicochemical property.

**Table 3:** Top-10 property patterns.

	**Pattern**	**Score**	**Significance**		**Pattern**	**Score**	**Significance**
**Hydrophobic**	100010.0.00	0.88	-25.622267	**Aliphatic**	111.0000010	0.90	-28.438190
			
	00011010.00	0.86	-29.127605		11100000010	0.89	-31.665638
			
	00011000111	0.86	-32.008152		111000.0010	0.89	-28.387123
			
	100010.000	0.85	-25.689735		111.00.0010	0.89	-25.159679
			
	10001000..0	0.85	-25.679390		101000101.0	0.88	-28.450298
			
	11101011000	0.85	-31.535349		1110001.1	0.87	-23.336674
			
	1000.00000	0.84	-26.223049		010.0010101	0.86	-28.455349
			
	1101011000	0.84	-28.097616		01..0010101	0.85	-25.175838
			
	0011010000	0.83	-29.159554		111.00100.0	0.85	-25.185696
			
	00.11010000	0.83	-29.148598		0100.001110	0.85	-28.344454

**Polar**	00011001000	0.87	-31.464071	**Aromatic**	11010..100	0.93	-23.787088
			
	00.11001000	0.86	-27.802887		1101...10	0.92	-17.538124
			
	00111001000	0.85	-31.448685		1101..010	0.91	-20.680222
			
	11000011000	0.84	-31.394987		1101.0.10	0.90	-20.681501
			
	00000000011	0.84	-31.472200		01.010.0010	0.87	-25.028446
			
	00101000001	0.83	-31.512842		010010100.0	0.88	-28.226509
			
	0001100.000	0.83	-27.699286		10010100.0	0.88	-25.118229
			
	11001000111	0.82	-31.390800		0.101000010	0.88	-28.193560
			
	11010000011	0.82	-31.397318		0.0.0010110	0.88	-24.919533
			
	110010.0111	0.81	-27.739485		01.010.001	0.87	-21.915537

**Small**	01011100101	0.84	-31.444056	**Positive**	110.10010	0.87	-23.358616
			
	11111101001	0.83	-31.323477		0.00011100	0.81	-24.565418
			
	10111100011	0.83	-31.408043		0000011100	0.80	-27.679506
			
	1011100101	0.82	-27.795000		---	---	---
			
	01101101001	0.82	-31.530676		---	---	---
			
	10111110100	0.81	-31.362995		---	---	---
			
	01101101.01	0.80	-27.816114		---	---	---
			
	0101110.101	0.80	-27.737080		---	---	---
			
	01101101101	0.80	-31.575062		---	---	---
			
	---	---	---		---	---	---

**Proline**	000001110.0	0.81	-29.866806	**Negative**	01.0.000011	0.80	-24.944452
			
	0000.1101	0.80	-23.843355		---	---	---
			
	000001101	0.80	-26.895651		---	---	---
			
	---	---	---		---	---	---
			
	---	---	---		---	---	---
			
	---	---	---		---	---	---
			
	---	---	---		---	---	---
			
	---	---	---		---	---	---

In Table 3 the retained property patterns are displayed using the binary representation; the occurrence of the respective physicochemical property in the pattern is denoted with an "1", whereas, "0" indicates that the position is occupied by an amino acid that does not share the respective property. Same as before, the "." is used to denote a position that can be occupied by an arbitrary residue, irrespective of its physicochemical classification.

An interesting remark is that for certain physicochemical properties (i.e. positive, negative), hardly any reliable associations are retained. Hence, we infer that patterns containing these properties are not significantly correlated with regions containing proline *cis *peptide bonds. This means that these properties are evenly distributed to the neighborhood of proline peptide bonds. Indeed, no association between the charge (either positive or negative) of the neighboring amino acids and the conformation of the peptide bond has been reported in the literature. In the case of prolines, we can see that in all three discovered patterns ("000001110.0", "0000.1101", "000001101") a triad of prolines, either successive or very close to each other, is maintained. Although, not identical, these patterns resemble the core pattern "P..P", frequently encountered in PRRs. Variations of this pattern are very common in peptide ligands, especially in proteins with SH3 and WW domains. The same situation was observed in the amino acid patterns, where some of the highest scoring patterns were populated mainly by two or three proline residues. However, the relatively low scores and the small number of retained associations, prevent us from attaching too much importance to the reported patterns.

**Figure 7:**
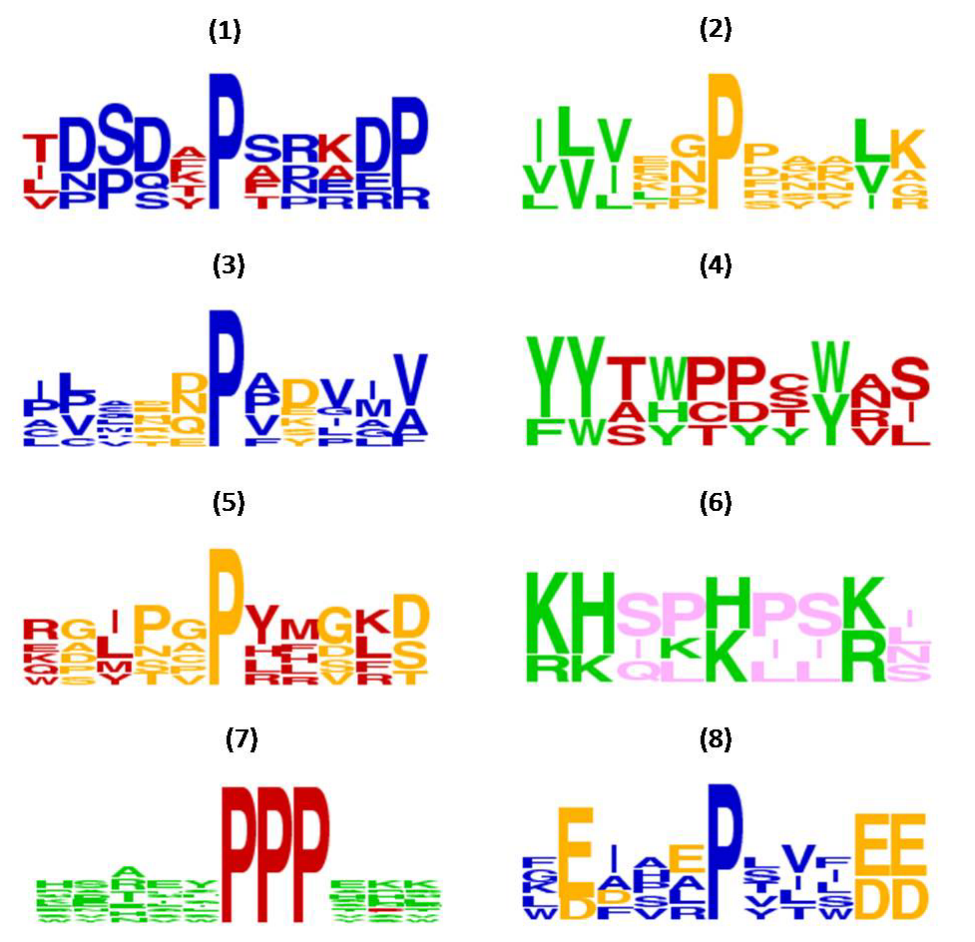
**Sequence logos for the most representative property patterns**. A sequence logo is provided for the highest scoring pattern of every property (Table 3). Different colors are used to discriminate between the amino acids that share or do not share the respective property. Specifically the highest scoring property patterns in the sequence logos are depicted in the following order: (1) hydrophobic, (2) aliphatic, (3) polar, (4) aromatic, (5) small, (6) positive, (7) proline, (8) negative.

As it is shown in Table 3, the highest scores are obtained when the aromatic character of the amino acids is under consideration; almost all retained patterns contain many aromatic residues (e.g. '11010..100', '1101...10', '1101..010', '1101.0.10'). Similar observations were made previously, concerning the amino acid patterns, where the aromatic residues occurred very frequently in the neighborhood of proline *cis *peptide bonds. The positive influence of aromatic amino acids is attributed to the C-H···π interaction or the stacking of the two rings [5]. However, there is no specific explanation for this in the literature and remains a matter of debate [5]. Moreover, high scores are also obtained when the hydrophobicity, the polarity or the aliphatic character of the amino acids are taken into account. Especially, in the case of aliphatic oriented patterns, a string of three consecutive aliphatic residues is observed. Most representative patterns are "111.0000010" and "101000101.0" as well as their variations. Furthermore, concerning polarity, clusters of two polar amino acids are frequently observed in the majority of the retained patterns. The most representative polar patterns are "00011001000", "11000011000", "00000000011", "11010000011" and their variations. Similar observations can be made in terms of hydrophobicity, where patterns of two consecutive hydrophobic residues are common among the highest scoring associations (e.g. "00011010.00", "00011000111", "11101011000", "1101011000", "0011010000"). Finally, in the case when small amino acids are concerned, although lower scores are obtained, it is clear that long stretches of small residues are quite frequent in regions containing *cis *peptide bonds. This is mostly observed in the patterns "11111101001", "10111100011", and "10111110100". In general, small residues are common in the neighborhood of *cis *peptide bonds, mainly due to the lack of steric hindrance [5]. The highest scoring pattern from each property is depicted as sequence logo [32] in Figure 7.

It should be highlighted that the significance measure for all retained patterns is very low, especially in the case of property patterns. Such low values for significance ensure that the derived associations are very unlikely to appear by chance, even in a very large biological database, and should in principle define highly reliable patterns.

All conclusions gained both from the amino acid and the property patterns are consistent with several findings, especially from recent studies [4,5,11], concerning the surrounding of *cis *conformations. In addition our study has discovered many previously unknown associations involved in the formation of the peptide bond, and in overall all these findings have been systematically formulated in the reported regular expression-type patterns.

### Functional classes

Once the patterns describing *cis *regions have been extracted, we aim to infer some qualitative conclusions about the functional classes they belong. Despite the increasing size of protein databases, the number of identified *cis *peptide bonds is still limited. Hence, the employment of patterns, which effectively and specifically generalize the existing knowledge about *cis *formations, can be proven quite profitable, since it allows for efficient extrapolation of gained conclusions in a wide range of protein molecules. Thus, systematic elicitation of the functional role of *cis *proline peptide bonds can be achieved.

**Figure 8:**
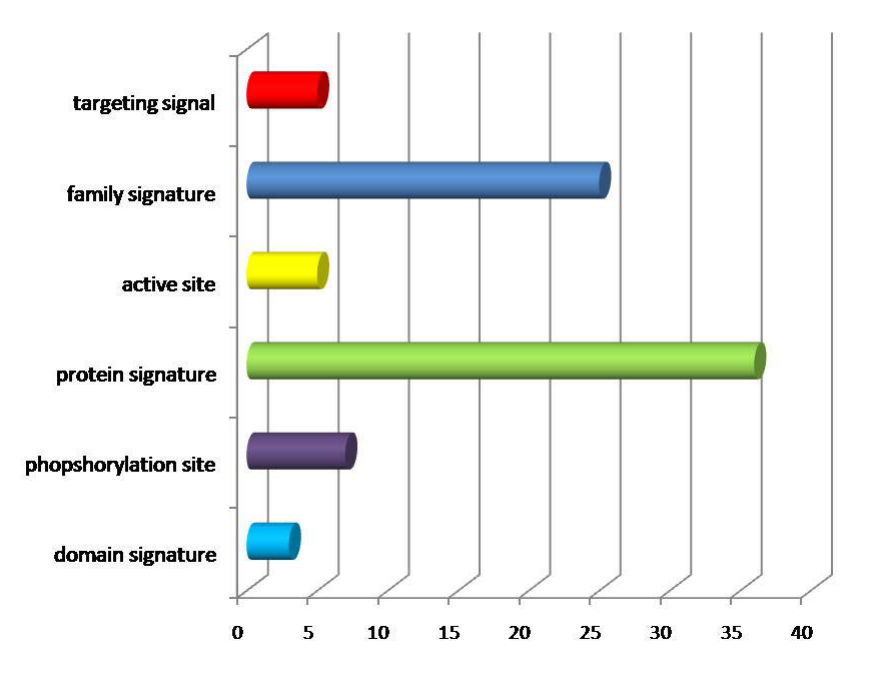
**Functional propensities of *cis *peptide bonds**. A significant correlation is observed for family and protein signatures. There is also noteworthy prevalence of *cis *regions with targeting signals, active and phosphorylation sites as well as domain signatures. The x-axis denotes the cumulative frequencies for every functional class.

The chart in Figure 8 depicts the functional groups that *cis *regions are associated with, along with indicative propensities for each group. The values on the x-axis refer to the cumulative number that a functional class was retrieved by the *cis *patterns. We observe a high propensity of *cis *regions towards protein and family signatures. Relatively lower propensities can be observed for targeting signals, active sites, phosphorylation sites and domain signatures. It is noteworthy that our analysis has rediscovered some functional associations already reported in the literature, thus, validating the current approach; in addition certain unknown associations have been suggested which prompt for further experimental exploration.

The functional prevalence of *cis *peptide bonds with certain core functionalities of the cell has been established in a variety of studies and is being further reinforced by the rationale of the PROSITE database construction. PROSITE contains protein segments grouped according to their function and sequence similarity. These segments have been better conserved than others during evolution and are very important for the function of the protein and/or for the maintenance of its three dimensional structure. Regarding the *cis *peptide bonds, it has been found that residues involved in their formation, are far more conserved than the rest of the residues which show the same extent of conservation as the whole protein. This observation highlights the significance of *cis *peptide bonds in protein structure and function during evolution, thus, enhancing the credibility of our findings.

The methodological analysis followed in the present study, can be generalized and utilized for the exploration of any important protein characteristic. Initially, from a set of unaligned protein sequences, a list of overrepresented patterns is extracted, which is further refined with the application of certain statistical criteria. In this sense, the nature and interactions in the neighborhood of any significant protein region can be investigated. Afterwards, the derived list of highly selective patterns can be functionally annotated by means of comparison against the records of the PROSITE or any similar biological database. The efficient exploitation of the information deposited in such repositories can systematically and reliably unravel the functional propensity of any protein motif.

## Conclusion

Although proline *cis/trans *isomerization is an active topic in the literature for many years, it has recently attracted considerable interest in order to uncover its mechanism and the factors that affect its occurrence. Proline *cis *peptide bonds are known to play a significant role in protein structure and function as well as implicate with the induction and progression of certain severe diseases. In this study, we perform a systematic analysis of the regions in proteins which contain *cis *proline peptide bonds. Several non-random both amino acid and property patterns are extracted which capture the nature and mechanism of *cis *prolyl conformations. Besides the qualitative conclusions about the nature of *cis *peptide bond, an extensive list of highly selective regular expression-type patterns is provided, which can also be helpful for quantitative assessment. Furthermore, the derived patterns are compared against the PROSITE records, in order to detect and localize the functional propensities of *cis *peptide bonds. Since such patterns, derived from the amino acid sequence can be characteristic of a certain protein family, it would be interesting to concentrate on a predefined number of protein families and aim to extract specific patterns for each family separately.

## Availability and requirements

To ensure reproducibility, all our datasets, results and analysis details, as well as links to relevant resources are available at the following URL: .

## Authors' contributions

KPE conceived, designed and implemented the study, working towards his PhD thesis. TPE and CP provided valuable comments and discussions. ANT and DIF supervised the study and provided substantial advice and guidance during all phases. All authors have read and approved the final manuscript.
